# Supplementary material for: Nutritional Supplementation for Obsessive-Compulsive Disorder: Protocol for a Systematic Review of Randomized Controlled Trials
Source: JMIR Res Protoc. 2025 Nov 18;14:e80240. doi: 10.2196/80240 (PMC12673304; doi:10.2196/80240)
Supplement: Multimedia Appendix 2 [file resprot_v14i1e80240_app2.docx]

| **Additional File 2**. Key terms for MEDLINE and EMBASE | | | |  |
| --- | --- | --- | --- | --- |
|  | | **Search** | **Query** |  |
| #1 | Diagnosis | | "obsessive-compulsive disorder"[mh] OR “obsessive-compulsive disorder"[tiab] |  |
| #2 | Nutritional supplements | | “5-HTP (5-hydroxytryptophan)”[tiab] OR “beta-carotene”[tiab] OR “biotin”[tiab] OR “calcium”[tiab] OR “choline”[tiab] OR “chromium”[tiab] OR “cobalamin”[tiab] OR “coenzyme Q10”[tiab] OR “fiber”[tiab] OR “fish oil”[tiab] OR “folate”[tiab] OR “folic acid”[tiab] OR “iron”[tiab] OR “L-arginine”[tiab] OR “L-tryptophan”[tiab] OR “magnesium”[tiab] OR “N-acetylcysteine”[tiab] OR “niacin”[tiab] OR “omega-3 fatty acids”[tiab] OR “omega-6 fatty acids”[tiab] OR “omega-7 fatty acids”[tiab] OR “niacin”[tiab] OR “niacin”[tiab] OR “pantothenic acid”[tiab] OR “potassium”[tiab] OR “probiotics”[tiab] OR “riboflavin”[tiab] OR “thiamin”[tiab] OR “vitamin A”[tiab] OR “vitamin B1”[tiab] OR “vitamin B12”[tiab] OR “vitamin B2”[tiab] OR “vitamin B3”[tiab] OR “vitamin B5”[tiab] OR “vitamin B6”[tiab] OR “vitamin B7”[tiab] OR “vitamin B9”[tiab] OR “vitamin C”[tiab] OR “vitamin D”[tiab] OR “vitamin E”[tiab] OR “vitamin K”[tiab] OR “zinc”[tiab] |  |
| #3 | Study type | | "randomized controlled trial"[pt] OR "controlled clinical trial"[pt] OR "randomized"[tiab] OR "randomly"[tiab] OR "trial"[tiab] | |
| #4 |  | | #1 AND #2 AND #3 | |
